# Transversus abdominis plane block provides effective and safe anesthesia in the cesarean section for an amyotrophic lateral sclerosis parturient: A case report

**DOI:** 10.1097/MD.0000000000027621

**Published:** 2021-10-29

**Authors:** Yun Wang, Yi Zhang, Shitong Li, Lianhua Chen, Jihong Jiang

**Affiliations:** Department of Anesthesiology, Shanghai General Hospital, Shanghai Jiao Tong University School of Medicine, Shanghai, P.R. China.

**Keywords:** amyotrophic lateral sclerosis, cesarean section, sevoflurane, transversus abdominis plane, volatile anesthesia

## Abstract

**Rationale:**

Amyotrophic lateral sclerosis (ALS) is a progressive neurodegenerative disease with the fatal course of muscle weakness. The published experience of anesthesia management in the cesarean section with ALS parturient is scant.

**Patient concerns:**

A 34-year-old woman was admitted to our center complaining of obvious dysphagia together with atrophy and weakness of quadriceps at 24 weeks of her pregnancy. Cesarean was planned at 36 weeks’ gestation due to the rapid deterioration of the mother.

**Diagnoses:**

The results of neurological examination, electromyography and spinal magnetic resonance imaging suggested ALS according to the EI Escorial World Federation of Neurology criteria.

**Interventions:**

Ultrasound-guided transversus abdominis plane block with 0.6 minimum alveolar concentration sevoflurane was used in this ALS parturient during her cesarean section procedure.

**Outcomes:**

This anesthesia strategy successfully met the demands of the surgery, helped avoid prolonged ventilation and prevent maternal respiratory complications.

**Lessons:**

Transversus abdominis plane block with subanesthetic concentrations of sevoflurane can provide effective and safe anesthesia in the cesarean section for a patient with ALS.

## 1 Introduction

Amyotrophic lateral sclerosis (ALS) is a relentlessly degenerative disease of the motor ganglia in the anterior horn of spinal pyramidal tracts.^[1]^ ALS is exceedingly rare in women of reproductive age, and pregnancy seems to do not have any effect on the onset of ALS.^[2]^ However, the clinical feature of ALS in parturient is overt due to the increase of progestogens activity.^[3]^ Cesarean delivery has been reported in ALS patients, but the published experience of anesthesia management is scant. We describe the anesthesia procedure of cesarean delivery of a woman developed ALS during pregnancy.

## 2 Case report

A 34-year-old woman, G4P3 (3 alive children), at the third month of her pregnancy this time, came to our hospital complaining of mild dysphonia and stiffness of gait. No neurologic disorders had been previously diagnosed in her family. The neurological examination showed hyperreflexia at the lower limbs and the electromyography provided evidence of acute and chronic denervation in active muscle, considering the neurodegenerative of the anterior horn cells of the spinal cord. Magnetic resonance imaging of the spine suggested degenerative diseases. The diagnosis was ALS under EI Escorial World Federation of Neurology criteria.^[4]^

The couple refused abortion confirmed by the gynecologist, and abandoned medical supervision. At 24 weeks, the patient developed obvious dysphagia together with atrophy and weakness of quadriceps, but no symptom of dyspnea appeared. However, due to the rapid deterioration of the mother, cesarean was planned primarily because of concerns about the potential maternal risk at 36 weeks’ gestation. The patient received a brief corticosteroid therapy aiming to accelerate the fetal lungs’ maturation. Ultrasound examination revealed that the fetus was in good condition and the estimated fetal body weigh was 3000 g. Pulmonary function tests showed a decrease in both forced vital capacity and forced expiratory capacity in the first second (73.2% and 75.1%, of predicted values) indicating a mild restrictive lung defect. There was no other abnormal physical or laboratory finding.

The cesarean procedure took place on Jun 22, 2018. Transversus abdominis plane (TAP) block combined with sub-anesthetic concentrations of volatile anesthesia was planned. A urinary catheter and a venous catheter were inserted in the preparation room. ECG monitoring, noninvasive arterial blood pressure, pulse oximetry, and capnometry were administered in the operation room. The mother's body weight was 57 kg and height was 157 cm.

**Figure 1:**
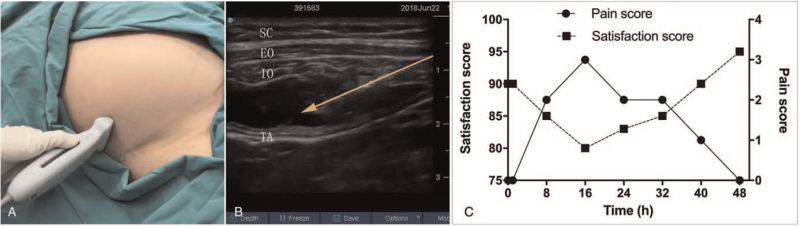
(A and B) Approach of ultrasound-guided lateral transversus abdominis plane (TAP) blocks. Orange arrow line indicates *the needle trajectory*. EO = external oblique, IO = internal oblique, SC = subcutaneous tissue, TA = transversus abdominis, TAP = transverse abdominis plane. (C) The outcome of pain and satisfaction score in actual patient data. The satisfaction score was evaluated according to the Leiden Perioperative Care Patient Satisfaction Questionnaire. Visual Analogue Scale (VAS) is recommended for the assessment of pain scores.

Before the surgery, bilateral ultrasound-guided TAP block was performed by an experienced anesthetist using 15 mL mixture of bupivacaine 0.375% and lidocaine 1% on each side (Fig. 1A and B). All the procedure was performed using aseptic technique. Fifteen minutes later, no movement and feeling in response to pain was tested in the area T9-T12 innervate. And, an incision infiltration with 0.375% bupivacaine was also used to perfect the analgesic effect. Before the operation start, the patient suddenly had anxiety and dysphoria. An intravenous bolus of ketamine (0.5 mg/kg) and propofol (1 mg/kg) was applied for sedation followed by the insertion of laryngeal mask to support the airway. During the procedure, anesthesia was supported by volatile anesthesia consisting of 1.5 vol% sevoflurane and 1 L/min oxygen (minimum alveolar concentration, minimum alveolar concentration [MAC] 0.6) administered by a volume-controlled anesthesia ventilator via the laryngeal mask. The obstetricians were satisfied with the anesthetic efficacy. Fifteen minutes after skin incision, a normal male infant weighting 2720 g (Apgar scores of 9 at 1 minute and 10 at 5 minutes) was born. Uterine contraction was good and the cesarean procedure lasted 30 minutes, with a total blood loss of 300 mL and fluid infusion of 1000 mL sodium lactate Ringer injection.

The patient became responsive 20 minutes after the discontinuation of the volatile anesthetic, and was able to breathe smoothly without the respirator. Because the patient was evaluated to be capable to protect her airway, the laryngeal mask was removed by the anesthetist (Table 1). Then she was delivered into the intensive care units. The pain scores of the mother were below 3 points in 48 hours after the surgery. The mother did not have any supplemental opioid analgesic requirement in the ICU and was quite satisfied with the quality of care (mean satisfaction score of 87.2%) according to the Leiden Perioperative Care Patient Satisfaction questionnaire (Fig. 1C). The patient discharged from our hospital 3 days after the surgery. The pulmonary function test 1 week after discharge did not show any worsen of the pulmonary function. At present the patient is still alive in a stable condition with dysarthria and weakness of the extremity. She was still able to walk with the aid of stick. Her son is in good health.

**Table 1:** Respiratory and circulatory variables during the surgery.

Statistic/moment	0 min	5 min	10 min	15 min	20 min	25 min	30 min	35 min	40 min	45 min	50 min	55 min	60 min	70 min
HR, bpm	100	115	102	96	89	86	104	89	85	88	86	88	84	82
BP, mmHg	120/76	125/75	124/80	118/58	109/57	105/54	110/67	100/47	120/52	125/57	104/56	110/67	108/65	100/43
SpO_2_ (%)	96	99	99	98	99	99	99	99	99	99	99	99	99	99
RR, breath/min	18	16	16	16	16	15	15	14	14	14	14	14	14	16

## 3 Discussion

Our report presents ultrasound-guided TAP block with subanesthetic concentrations of volatile anesthesia provides effective and safe anesthesia for cesarean section in a parturient with ALS. Our team chose the TAP block with subanesthetic concentrations of volatile anesthesia instead of neuraxial anesthesia as the primary strategy not only considering the preexisting low back pain of the parturient, but also the increased susceptibility of spinal cord cells to the potential neuro toxic effects of local anesthetics and the needle injury, both may risk maternal survival time after the delivery.

Although vaginal and cesarean delivery have been both reported in ALS patient,^[5]^ cesarean section may be the best choice. The probability of assisted delivery was greatly increased due to the weakness of the profound motor weakness. Also, due to progressive atrophy and weakness of respiratory muscles, parturient with ALS may not be able to increase breathing appropriately to meet the oxygen demand during labor.^[6]^

General anesthesia can also be chosen for caesarean delivery, but has its risks in ALS patient population. Muscle relaxant is the first question we should think about in general anesthesia procedure, which may cause respiratory dysfunction and prolonged ventilation because of susceptibilities of parturient with ALS.^[7]^ Xiao et al^[6]^ and Jiao et al.^[8]^ reported successful cases which performed total intravenous anesthesia without muscle relaxant for parturient with ALS undergoing cesarean section. Slight volatile anesthesia of 1.5 vol% sevoflurane (MAC 0.6) was inhaled in our case instead of propofol based total intravenous anesthesia mainly concerning the potential maternal and fetal risks caused by high doses of propofol. Sevoflurane recommend as a inhalation agent with rapid onset and recovery is safe for mother and newborn of general anesthesia during cesarean section.^[9]^

TAP block was often applied as a regional anesthesia technique before the surgery for post-cesarean delivery analgesia. The ultrasound-guided injection of local anesthetics targeting the nerve roots of T9-L1 can provide a quite satisfied prepare for the lower abdominal procedures. Using an ultrasound-guided technique, the needle path can be in-plane with the plane of the ultrasound which may aid accuracy and safety.^[10]^ Regional anesthesia offers various advantages because its ability to provide sustained pain relief and to protect bulbar function. The dosing strategy of mixture of bupivacaine 0.375% and lidocaine 1%, 15 mL per side mainly block the sensory nerves, but may not affect the motor nerve causing the worsen of the respiratory function. It is paramount to choose TAP block to help to relieve the pain intra and post-operatively. And TAP block can also reduce the risks of delayed respiratory depression due to rostral spread of hydrophilic opioids like morphine and adverse effects on the newborn to a large extent.^[11,12]^

Considering that TAP block may have no effect on visceral pain, subanesthetic concentrations of volatile anesthesia (MAC 0.6) was used to block visceral pain and reverse rapidly. We used laryngeal mask to support the airway to meet the increasing oxygen consumption during the delivery. Laryngeal mask as the second-generation supraglottic airway device with the benefits of less stress response^[13]^ and better physical and psychological comforts^[14]^ is safe in general anesthesia for cesarean section without severe fetal compromise,^[15]^ and it is easier to insert and well tolerated in spontaneous breathing without muscle relaxant than tracheal intubation. Because of the enough time of fasting and water deprivation of this patient, the laryngeal mask provided a safe airway and was removed quickly after operation without causing pulmonary aspiration in our case. Stable respiratory function after surgery to avoid additional respirator support is also crucial for the outcome of ALS patient.^[16]^ In brief, the bilateral TAP block with slight volatile anesthesia provides a smooth recovery after the surgery and a satisfying experience of perioperative period. Furthermore, the pulmonary function test showed no obvious decrease 1 week later, and both the mother and baby are in a stable condition now.

## 4 Conclusions

This case presents a use of TAP block with subanesthetic concentrations of volatile anesthesia for cesarean section in a parturient with amyotrophic lateral sclerosis at 36 weeks’ gestation. This anesthesia strategy successfully met the demands of the surgery, helped avoid prolonged ventilation and prevent maternal respiratory complications. However, the efficacy of our strategies may be affected by selection bias because of the nature of case report. More report and research are still warranted.

## Author contributions

**Data curation:** Yi Zhang, Jihong Jiang.

**Funding acquisition:** Jihong Jiang.

**Investigation:** Yi Zhang, Lianhua Chen.

**Methodology:** Shitong Li.

**Project administration:** Yi Zhang, Jihong Jiang.

**Supervision:** Shitong Li, Lianhua Chen, Jihong Jiang.

**Writing – original draft:** Yun Wang.

**Writing – review & editing:** Yun Wang, Shitong Li, Jihong Jiang.

## References

[1] Kawamichi Y Makino Y Matsuda Y Miyazaki K Uchiyama S Ohta H. Riluzole use during pregnancy in a patient with amyotrophic lateral sclerosis: a case report. J Int Med Res 2010;38:720–6. 20515588 10.1177/147323001003800237

[2] Tyagi A Sweeney BJ Connolly S. Amyotrophic lateral sclerosis associated with pregnancy. Neurol India 2001;49:413–4. 11799421

[3] Rooney JPK Visser AE D’Ovidio F. A case-control study of hormonal exposures as etiologic factors for ALS in women: Euro-MOTOR. Neurology 2017;89:1283–90. 28835399 10.1212/WNL.0000000000004390

[4] Brooks BR Miller RG Swash M Munsat TL. World Federation of Neurology Research Group on Motor Neuron D. El Escorial revisited: revised criteria for the diagnosis of amyotrophic lateral sclerosis. Amyotroph Lateral Scler Other Motor Neuron Disord 2000;1:293–9. 11464847 10.1080/146608200300079536

[5] Sarafov S Doitchinova M Karagiozova Z. Two consecutive pregnancies in early and late stage of amyotrophic lateral sclerosis. Amyotroph Lateral Scler 2009;10:483–6. 19922145 10.3109/17482960802578365

[6] Xiao W Zhao L Wang F Sun H Wang T Zhao G. Total intravenous anesthesia without muscle relaxant in a parturient with amyotrophic lateral sclerosis undergoing cesarean section: A case report. J Clin Anesth 2017;36:107–9. 28183545 10.1016/j.jclinane.2016.10.009

[7] Rosenbaum KJ Neigh JL Strobel GE. Sensitivity to nondepolarizing muscle relaxants in amyotrophic lateral sclerosis: report of two cases. Anesthesiology 1971;35:638–41. 5124745 10.1097/00000542-197112000-00017

[8] Li J Zeng H Li M Wang J. Anesthetic management of a parturient with amyotrophic lateral sclerosis undergoing cesarean section. Chin Med J (Engl) 2020;133:1371–2. 32398520 10.1097/CM9.0000000000000809 PMC7289292

[9] Devroe S Van de Velde M Rex S. General anesthesia for caesarean section. Curr Opin Anaesthesiol 2015;28:240–6. 25827280 10.1097/ACO.0000000000000185

[10] Jadon A Jain P Chakraborty S. Role of ultrasound guided transversus abdominis plane block as a component of multimodal analgesic regimen for lower segment caesarean section: a randomized double blind clinical study. BMC Anesthesiol 2018;18:53. 29759061 10.1186/s12871-018-0512-x PMC5952861

[11] Belavy D Cowlishaw PJ Howes M Phillips F. Ultrasound-guided transversus abdominis plane block for analgesia after Caesarean delivery. Br J Anaesth 2009;103:726–30. 19700776 10.1093/bja/aep235

[12] Tran TM Ivanusic JJ Hebbard P Barrington MJ. Determination of spread of injectate after ultrasound-guided transversus abdominis plane block: a cadaveric study. Br J Anaesth 2009;102:123–7. 19059922 10.1093/bja/aen344

[13] Nekhendzy V Ramaiah VK Collins J Lemmens HJ Most SP. The safety and efficacy of the use of the flexible laryngeal mask airway with positive pressure ventilation in elective ENT surgery: a 15-year retrospective single-center study. Minerva Anestesiol 2017;83:947–55. 28358175 10.23736/S0375-9393.17.11403-3

[14] Wang ZP Ma J Wang S Yu LN Wei JF Xu JD. [Application of sevoflurane and laryngeal mask in cesarean section in women with heart disease]. Nan Fang Yi Ke Da Xue Xue Bao 2018;38:229–33. 29502065 10.3969/j.issn.1673-4254.2018.02.18 PMC6743878

[15] Fernandes NL Dyer RA. Anesthesia for Urgent Cesarean Section. Clin Perinatol 2019;46:785–99. 31653308 10.1016/j.clp.2019.08.010

[16] Kock-Cordeiro DBM Brusse E van den Biggelaar RJM Eggink AJ van der Marel CD. Combined spinal-epidural anesthesia with non-invasive ventilation during cesarean delivery of a woman with a recent diagnosis of amyotrophic lateral sclerosis. Int J Obstet Anesth 2018;36:108–10. 30017643 10.1016/j.ijoa.2018.06.001

